# Results from the first culturally tailored, multidisciplinary diabetes education in Lebanese adults with type 2 diabetes: effects on self-care and metabolic outcomes

**DOI:** 10.1186/s13104-022-05937-0

**Published:** 2022-02-10

**Authors:** Ola Sukkarieh-Haraty, Leonard E. Egede, Georges Khazen, Joelle Abi Kharma, Natali Farran, Maya Bassil

**Affiliations:** 1 Alice Ramez Chagoury School of Nursing, Lebanese American University, Byblos, Lebanon; 2 Center for Advancing Population Science, Medical College of Wisconsin, Milwaukee, USA; 3 Department of Computer Sciences and Mathematics, Lebanese American University, Byblos, Lebanon; 4 Department of Natural Sciences, School of Arts and Sciences, Lebanese American University, Beirut, Lebanon; 5 Hariri School of Nursing, American University of Beirut, Beirut, Lebanon; 6 Department of Human Nutrition, College of Health Sciences, QU Health, Qatar University, P.O. Box 2713, Doha, Qatar

**Keywords:** Diabetes self-care, Culture, Multidisciplinary intervention, Education, Lebanon, Type 2 diabetes

## Abstract

**Objective:**

Diabetes self-management education (DSME) is an essential component of lifestyle management needed for diabetes care. This pilot-study tested the effect of culturally-tailored education targeting diabetes selfcare on glycemia and cardiovascular risk factors of Lebanese with type 2 diabetes mellitus (T2DM) (n = 27; Age: 61 ± 10 yrs, 59% males, HbA1c: 8.98 ± 1.38%).

**Results:**

Diabetes self-care (Diet, Self-Monitoring Blood Glucose and foot care) improved after 6 months, which was reflected in a significant drop in glycemic levels (HbA1c:-0.5%; FPG: − 38 mg/dl), and cholesterol/HDL ratio (4.45 ± 1.39 vs. 4.06 ± 1.29). Waist circumference decreased at 6 months compared to 3 months (p < 0.05). This is the first effective culturally-tailored intervention that improved self-care, glycemic control, body adiposity and lipid profile of Lebanese with T2DM. Larger scale implementation with representative sample is warranted.

**Supplementary Information:**

The online version contains supplementary material available at 10.1186/s13104-022-05937-0.

## Introduction

Diabetes self-care includes medication adherence, exercise, healthy nutrition, self-monitoring of blood glucose (SMBG), and foot care [1]. Effectively, diabetes self-care education has been shown to improve glycemic levels, dietary habits, body anthropometrics (weight, body mass index and waist circumference) [2], and lipid profile [3]. The American Association of Diabetes Educators (AADE) guidelines recommend cultural tailoring of educational program, taking into consideration ethnic and cultural beliefs and practices to ensure optimal self-care adherence and glycemic outcomes [4]. One important cultural barrier that is commonly reported to impede diabetes self-care behavior is diabetes fatalism, which is described as “a complex psychological cycle characterized by perceptions of despair, hopelessness, and powerlessness” [5]. Specifically, diabetes fatalism is linked to inadequate medication adherence, poor diet and lifestyle behaviors, uncontrolled glycemic levels, and decreased quality of life [5–7].

Lebanon is a country in the Middle East where diabetes prevalence has been increasing over the years, and it is currently estimated at 12.2% [8]. The Lebanese population with type 2 diabetes mellitus (T2DM) also has a high prevalence of cardiovascular risk factors such as smoking, obesity, and physical inactivity [9]. Younger and heavier Lebanese T2DM patients with lower levels of education and no diabetes co-morbidities were shown to exhibit more fatalistic attitudes and uncontrolled glycemic levels [10, 11]. Lebanese patients T2DM demonstrated low levels of physical exercise [12] and the majority do not receive complete therapeutic management recommended for patients on chronic medication [13]. A sole recent randomized control trial found improvement in diabetes self-care activities and glycemic outcomes, 3 months after the intervention compared to baseline [14]. The intervention included multidisciplinary patient education and included Diabetes self-management education (DSME), but it did not address cultural barriers.

Diabetes education programs that targets self-care in Lebanon are scarce. This pilot study aims to investigate the effectiveness of a diabetes educational program on diabetes self-care, glycaemia, and cardiovascular risk factors in a Lebanese population with T2DM.

## Main text

### Methods

#### Sample

This study has quasi experimental design. A convenience sample of adults with T2DM, on oral anti-hyperglycemic agents, was recruited from two primary health care centers (PHCs) in Beirut. Inclusion criteria included being Lebanese, aged 18 years or older, diagnosed with T2DM for at least 1 year, on oral hypoglycemic therapy, and having an HbA1c level above 7.5% within the past 3 months. A total of 28 subjects were required to achieve a power of 80% and a level of significance of 5% (two sided) [15], with an effect size d = 0.5 and standard deviation SD = 2.11 for HbA1c [16].

#### Data collection

The participants completed the following previously validated instruments in the participants’ language (Arabic):

##### Summary of diabetes self-care activities scale (SDSCA)

SDSCA consists of 12 items that assess diabetes self-care, divided into four self-care activities: diet, exercise, foot care, self-monitoring of blood glucose and diabetes medication taking. SDSCA is scored by taking the raw score from each set of self-care tasks and converting it to a standardized score with a mean of zero and standard deviation of 1 [17]. Comparable to the original version, the Arabic version [18] revealed average inter-item reliability ranged between − 0.11 and 0.79.

##### Diabetes knowledge test (DKT)

DKT is a 23-item scale that measures diabetes knowledge. It is divided into two sections: 14-item general test and 9-item insulin scale. The highest score is 23 points and lowest is zero, whereby higher scores designate better knowledge of diabetes and its care [19]. In an Arabic speaking adolescent population, reliability analyses of the 2 subscales and the total resulted in 0.3, 0.25, and 0.49, respectively [20]. We used the 14-item general test since selected participants were on oral hypoglycemic treatment.

##### Diabetes fatalism scale (DFS)

DFS is a 12-item scale that measures diabetes fatalism. The higher the score of the scale, the greater the individual’s belief in diabetes fatalism would be [5]. The authors translated and back translated the instrument to and from Arabic and the resulting Arabic scale revealed a Cronbach’s alpha of 0.77 in the present study, which is comparable to the original version’s Cronbach’s alpha of 0.804.

##### Social support scale (SS)

The literature documents that the greater the social support for patients with diabetes, the better the motivation to adhere to their diabetes self-care, thereby resulting in well-controlled glycemic levels (A1C) [21]. The present intervention targeted the affective domain that favors positive attitudes towards the desirable health behavioral outcome and mobilizing the social support network as to optimize motivation. The Social Support Scale used consists of 12 items that measure social support received and social attitudes [22]. Each item is measured on 5-point Likert Scale from strongly disagree to strongly agree. The higher the cumulative score, the better the support by family and friends about diabetes and its treatment. The scale has been translated to Arabic and used with a Lebanese population with type 2 diabetes and resulted in Cronbach’s alpha of 0.88 [23].

##### Demographic information

Demographic characteristics (gender, age, marital status, education, employment status, income, and medical insurance coverage) and health-related characteristics (weight, height, body mass index BMI, diabetes duration, treatment, family history, complications and smoking status) were collected.

##### Clinical outcomes

1. Glycemic control and lipid profile

Blood samples were drawn to measure HbA1c, Fasting Plasma Glucose (FPG) and lipid profile (Total-Cholesterol, LDL-C, HDL-C and Triglycerides) using international standard procedures [24]. Glycemic control was measured by HbA1c and FPG and cardiovascular risk factors were measured by lipid profile.

FPG were identified as measures of glycemic control.

2) Anthropometric measurements

Weight (kg), height (cm) and waist circumference (cm) were collected using standard procedures [25], while BMI was computed (kg/m^2^). Body composition including body fat mass (kg) and fat free mass (kg) was measured using bioelectrical impedance (Tanita BC-418, Tanita Corporation, Tokyo, Japan).

#### Procedures

##### Recruitment

The researchers obtained approval from the Institutional Review Board (IRB) of the academic institution and the directors of the PHCs to conduct the study. The researchers informed the general practitioners and the administrative officer in the PHCs about the study and the inclusion criteria of the participants. Based on administrative officers reviews, the researcher contacted the selected participants during their clinical visits in the waiting room and informed them about the study. Interested participants were taken to a private room and asked to give their informed consent (Additional file 1: Fig. S1).

##### Description of the intervention

Based on ADA’s National Standards for Diabetes Self-Management Education and Support [26], two educational sessions (4 h each) were administered at the PHC for a group of 10 patients over 2 days. The sessions covered pathophysiology of diabetes, diet, physical exercise, self-monitoring of blood glucose, adherence to medication, foot care, diabetes complications and psychosocial issues, mainly fatalism that is pertinent to the Lebanese culture. The education program was interdisciplinary. The Nurse Diabetes Educator provided skills for adequate foot care, glucose self-monitoring and medication adherence. The Dietitian Diabetes Educator covered portion sizes and the different food groups and explained the importance of exercise. USDA My-plate tool was used, as it is an easy-to-grasp representation of relative portion sizes for different food groups. It was tailored based on participants’ food affordability and norms of traditional cooking. Exercise was based on understanding of participants environmental facilitators and barriers as well as encouraging women’s engagement given their overwhelming role of being primary care givers. The social worker, recruited from the PHC, had profound understanding of the participants’ problems, backgrounds, beliefs and systems values especially regarding fatalism. The social worker addressed fatalism and its effect on diabetes self-care contextually; diabetes related myths and misconceptions were addressed. Patients were encouraged to bring their spouses to the education sessions and were instructed to keep daily logs of glucose self-monitoring. Every patient was provided a personalized diet based on his/her needs and asked to adhere to it for the duration of the study. Data collection consisted of surveys (aforementioned), fasting blood samples (HbA1c, Fasting Plasma Glucose, lipid profile) and anthropometric measurements (weight, height, BMI, waist circumference & body composition) collected on the day of the intervention (baseline), 3 months and 6 months, post-intervention. Following the intervention, researchers conducted phone calls using a phone script, on monthly basis until the end of study (6 months) to provide guidance and support where needed, and ensure adherence.

#### Data analysis

Descriptive statistics were computed for the surveys, with means and standard deviations (SD) for continuous variables and percent for nominal variables. Paired t-test was used to compare continuous variables between baseline and 6 months, while repeated measures ANOVA was used for comparisons at 3 different time points. Post-hoc Tukey’s test was computed when significance was obtained. Level of significance was set as p < 0.05. SPSS version 23 was used to analyze the data.

### Results

Demographic and health related -characteristics are presented in Table 1.

**Table 1:** Characteristics of study participants

	*M*	*SD*
Age	61.9	8.2
Number of comorbid conditions	2.2	1.6

	N	%
Gender		
Male	16	59.3
Marital status		
Married	23	85.2
Unmarried	4	14.8
Level of education		
< 11 years	15	55.6
High school and above	12	44.4
Occupation		
Employed	6	22.2
Unemployed	21	77.8
Insurance		
Yes	14	51.9
Income		
Have enough to make ends meet	8	29.6
Do not have enough to make ends meet	19	70.4
Diabetes duration		
≤ 10 years	11	40.7
> 10 years	16	59.3
Family history		
Yes	20	74.1
Previous education about diabetes		
Yes	19	70.4
Smoking		
Yes	12	44.4

Data is presented as mean and standard deviation (SD) for continuous variables and N and percentage (%) for categorical variables

At the end of the study (6-month post-intervention), both HbA1c and fasting glucose decreased by 4% and 19%, respectively (Table 2). Regarding Diabetes self-care activities, significantly (p < 0.05) higher scores for diet (5.00 vs. 2.38), SMBG (5.15 vs. 1.61) and foot care (5.48 vs. 3.56) were obtained at 6 months compared to baseline (Table 1). Social support scores increased slightly (46.37 vs. 39.63, p = 0.07) (Table 2).

**Table 2:** Glycemic control, diabetes knowledge, self-care, fatalism, and social support at baseline and 6 months, post intervention

	Baseline		6 months		Mean difference	95% CI for Mean difference	p-value
	M	SD	M	SD			
Glycemic control							
HbA1c %	8.98	1.38	8.63	1.11	− 0.35	− 0.98; 0.26	0.24
FPG mg/dl	203.96	69.14	165.33	53.98	− 38.63	− 70.42; − 6.84	0.02*
DKT score	8.14	2.19	8.44	2.37	0.3	− 0.85; 1.45	0.61
SDSCA scores							
General Diet	2.38	2.35	5.00	2.43	2.62	1.33; 3.91	0.0003**
Exercise	2.52	2.48	3.11	2.04	0.58	− 0.62; 1.81	0.32
SMBG	1.61	2.24	5.15	1.98	3.54	2.33; 4.76	< 0.001**
Foot care	3.56	3.14	5.48	2.75	1.92	0.32; 3.52	0.02*
Medication compliance	6.48	1.81	7	0	0.52	− 0.22; 1.26	0.16
DFS score	27.59	7.83	26.31	8.02	− 1.28	− 3.90; 1.52	0.373
SS score	39.63	13.59	46.37	13.28	6.74	− 0.56; 14.05	0.069

Data is presented as mean and standard deviation (SD); *p < 0.05 **p < 0.001 (paired t-test). FPG: Fasting plasma Glucose; DKT: Diabetes Knowledge Test; SDSCA: Summary of Diabetes SelfCare Activities; SMBG: self-monitoring of blood glucose; DFS: diabetes fatalism scale; SS: Social Support Scale

Table 3 reports anthropometric measurements and lipid profile at baseline and 6 months postintervention. Ratios of Total Cholesterol/HDL-C and LDLC/HDL-C were significantly (p < 0.05) improved at 6 months post-intervention, which was mainly driven by an increase in HDL-C (45.59 vs. 42.91, p = 0.1) (Table 3).

**Table 3:** Anthropometric parameters and blood lipid profile at baseline and 6 months, post intervention

	Baseline		6 months		Mean difference	95% CI for mean difference	p-value
	M	SD	M	SD			
Anthropometric parameters							
Weight kg	81.41	18.49	81.70	19.43	0.29	− 2.08; 0.32	0.15
BMI kg/m^2^	31.28	5.47	31.00	5.66	− 0.28	− 0.76; 0.20	0.24
Waist circumference cm	107.52	12.91	106.59	12.01	− 0.93	− 3.24; 1.39	0.42
Fat %	35.19	7.03	34.53	5.99	− 0.66	− 2.11; 0.82	0.37
Fat mass kg	30.86	11.53	29.89	10.09	− 0.97	− 2.41; 0.47	0.18
Fat free mass kg	53.93	9.36	54.5	10.62	0.57	− 0.90; 2.04	0.44
Lipid profile							
Triglycerides mg/dl	181.96	90.28	177.59	96.12	− 4.37	− 32.74; 23.99	0.75
Total cholesterol mg/dl	182.03	37.44	175.96	31.27	− 6.07	− 18.86; 6.71	0.34
HDL-C mg/dl	42.91	10.21	45.59	10.13	2.68	− 0.6; 5.97	0.11
Total-C/HDL-C	4.44	1.39	4.06	1.29	− 0.38	− 0.73; − 0.06	0.02*
LDL-C mg/dl	107.48	32.38	102.63	27.00	− 4.85	− 15.03; 5.32	0.34
LDL-C/HDL-C	2.64	1.07	2.37	.84	− 0.27	− 0.53; − 0.003	0.047*

Data is presented as mean and standard deviation (SD); *p < 0.05 paired t-test; BMI: Body mass index; Total-C: Total cholesterol; HDL-C: High-density lipoprotein cholesterol; LDL-C: Low density lipoprotein cholesterol

Improvements in glycemic control (HbA1c and FPG), diet and SMBG were evident at 3 & 6-month post-intervention (p < 0.05 vs. baseline). Foot care improved at 6 months (p < 0.05 vs. baseline). Waist circumference decreased at 6 months compared to 3 months (p < 0.05) but not to baseline, while Total Cholesterol/HDL-C and LDL-C/HDL-C improved only at 6 months compared to baseline (Additional file 2: Fig. S2).

### Discussion

The improvement in glycemic control was not only statistically but also clinically significant [27]. Specifically, HbA1c was decreased by more than 0.5% at 3 months, post intervention and this was maintained at 6 months concurrent with studies conducted on other populations [3, 6].

#### Diabetes self-care

Self-care is not adopted at the same level in all areas [28]. Our findings suggest that patients adhered early on to the diabetes regimen that was tailored individually to their anthropometric measures, glucose and lipid profile. Findings are comparable to local [14] and regional studies [16]. Self-monitoring of blood glucose (SMBG) improved substantially in our intervention. Glucose self-monitoring may ensure involvement of the patient in controlling their glycemia, hence shifting more responsibility to the patient [29]. Additionally, SMBG shows real-time results about glycemic levels, enabling for informed alterations in lifestyle and medication.

Amelioration in foot care was only evident at 6 months, post intervention. On the other hand, exercise did not improve, which is comparable to an interventional study delivered to Emirati patients with T2DM [16]. No change was observed in medication adherence since baseline scores were high (6.48 over 7) indicating that participants were already adherent to the medication intake.

#### Diabetes knowledge

The reported improvement in some diabetes self-care activities was not paralleled by an increase in diabetes knowledge from baseline values. Behavioral research has well established that knowledge does not guarantee practice. Indeed, diabetes knowledge is not the sole predictor of self-care behaviors [6, 7].

#### Diabetes fatalism

Diabetes fatalism did not change from baseline, which might indicate that fatalism in this population is a constant trait (as opposed to a state like depression), a culturally grounded belief that is non-modifiable by a single intervention [5]. Diabetes fatalism is widespread in Lebanon and is associated with poor glycemic control [10, 11]. Alternatively, involving religious authorities in the education program, may ameliorate fatalism more effectively since it is closely related to religious beliefs among Arabs [30].

#### Lipid profile

The present intervention resulted in statistically significant differences in total cholesterol/HDL-C as well as LDL-C/HDL-C ratio post 6 months, concurrent with a decrease in waist circumference. Despite early adherence to diet, favorable effects on lipid profile were only evident at 6 months, the time it took for abdominal obesity to improve. Our results are consistent with randomized controlled studies conducted in Jordan [31] and in Qatar [32] that revealed statistically significant reduction in BMI 1 year post-intervention.

## Limitations

Despite the promising results shown in the current study, there are also limitations to note, hence we provide interpretations of findings with reservations. First, the study is a pilot quasi-experimental design, hence participants were not randomized and there was no control group. Therefore, we cannot establish thoroughly the impact of the study. Additionally, the sample was recruited from two PHCs only, thus the generalizability of our findings to other health care settings and all Lebanese patients with T2DM still needs to be investigated.

## Supplementary Information

**Additional file 1: Figure S1.** Patient recruitment.

**Additional file 2: Figure S2.** SDSCA parameters, glucose control, waist circumference and blood lipids at baseline, 3 months and 6 months, post-intervention.

## Data Availability

The data and materials generated and/or analyzed during the current study are available from the corresponding author on reasonable request.

## Abbreviations

AADE: American Association of Diabetes Educators
ADA: American Diabetes Association
BMI: Body mass index
DFS: Diabetes Fatalism Scale
DKT: Diabetes Knowledge Test
DSME: Diabetes self-management education
FPG: Fasting plasma glucose
HbA1c: Glycosylated hemoglobin
IRB: Institutional review board
PHC: Primary health care center
SD: Standard deviation
SDSCA: Summary of Diabetes Self-Care Activities
SMBG: Self-monitoring of blood glucose
SS: Social Support
T2DM: Type 2 diabetes mellitus
