# Supplementary figures and images for: Results from the first culturally tailored, multidisciplinary diabetes education in Lebanese adults with type 2 diabetes: effects on self-care and metabolic outcomes
Source: BMC Res Notes. 2022 Feb 10;15:39. doi: 10.1186/s13104-022-05937-0 (PMC8832854; doi:10.1186/s13104-022-05937-0)

**Figure S1: Patient Recruitment**


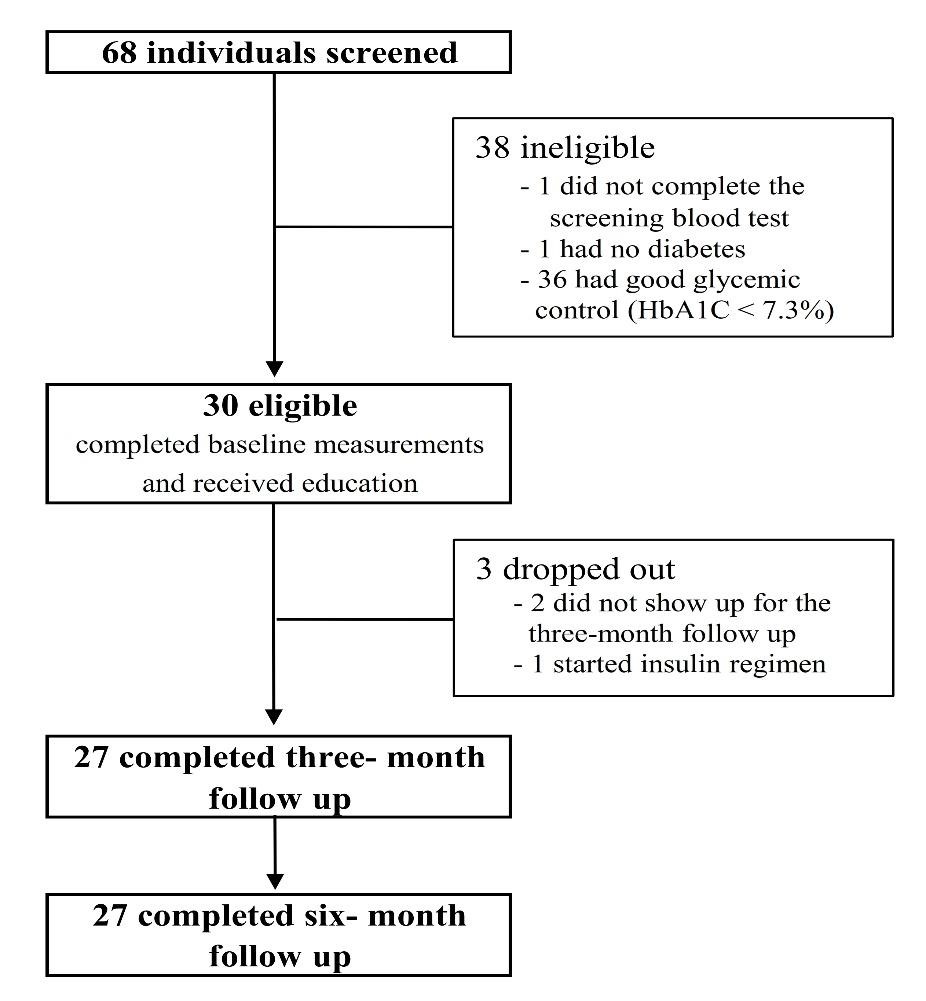

Supplement: Supplementary file 1 — Additional file 1: Figure S1. Patient recruitment. [file 13104_2022_5937_MOESM1_ESM.docx]
