# Supplementary material for: Results from the first culturally tailored, multidisciplinary diabetes education in Lebanese adults with type 2 diabetes: effects on self-care and metabolic outcomes
Source: BMC Res Notes. 2022 Feb 10;15:39. doi: 10.1186/s13104-022-05937-0 (PMC8832854; doi:10.1186/s13104-022-05937-0)
Supplement: Supplementary file 2 — Additional file 2: Figure S2. SDSCA parameters, glucose control, waist circumference and blood lipids at baseline, 3 months and 6 months, post-intervention. [file 13104_2022_5937_MOESM2_ESM.docx]

**Figure S2:** SDSCA parameters, glucose control, waist circumference and blood lipids at baseline, 3 months and 6 months, post-intervention.


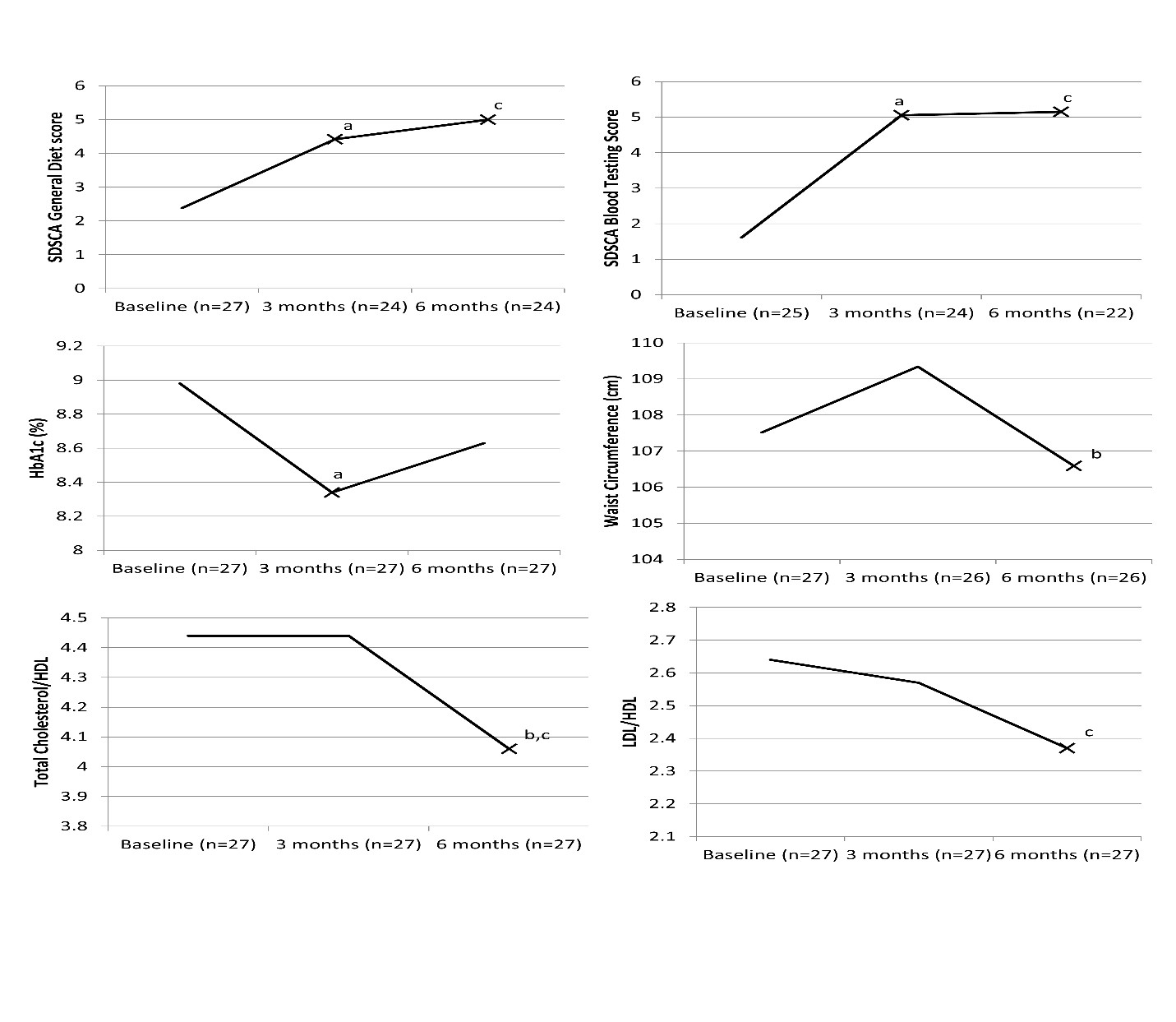
 ^a^ significant change between baseline and 3 months, ^b^ significant change between 3 and 6 months, ^c^ significant change between baseline and 6 months (Repeated measure-ANOVA with tukey’s posthoc test)
